# Incidence of biomarkers in high-grade gliomas and their impact on survival in a diverse SouthEast Asian cohort - a population-based study

**DOI:** 10.1186/s12885-020-6536-x

**Published:** 2020-01-31

**Authors:** Samantha Ya Lyn Ang, Lester Lee, Angela An Qi See, Ting Yao Ang, Beng Ti Ang, Nicolas Kon Kam King

**Affiliations:** 1 Department of Neurosurgery, National Neuroscience Institute, 11 Jalan Tan Tock Seng, Singapore, 308433 Singapore; 2 Department of Neurosurgery, Singapore General Hospital, Outram Rd, Singapore, 169608 Singapore; 3 Duke-NUS Medical School, 8 College Rd, Singapore, 169857 Singapore

**Keywords:** High-grade glioma, Incidence, MGMT, 1p19q, IDH, ATRX, Asian

## Abstract

**Background:**

Gliomas consist of a heterogeneous group of tumors. This study aimed to report the incidences of O^6^-methylguanine-DNA-methyltransferase (MGMT) promoter methylation, 1p19q co-deletion, isocitrate dehydrogenase (IDH) gene mutations, and inactivating mutations of alpha-thalassemia/mental retardation syndrome X-linked (ATRX) in high-grade gliomas in an ethnically diverse population.

**Methods:**

Records of patients who underwent surgery for high-grade gliomas from January 2013 to March 2017 at our institution were obtained. The patients’ age, gender, ethnicity, Karnofsky Performance Scale (KPS) score, ability to perform activities of daily living (ADLs), tumor location and biomarkers status were recorded. Data were analyzed using chi-square and Mann-Whitney U tests, Kaplan-Meier estimates and log-rank test.

**Results:**

181 patients were selected (56 with grade III gliomas, 125 with grade IV gliomas). In the grade III group, 55% had MGMT promoter methylation, 41% had 1p19q co-deletion, 35% had IDH1 mutation and none had ATRX loss. In the grade IV group, 30% had MGMT promoter methylation, 2% had 1p19q co-deletion, 15% had IDH1 mutation and 8% had ATRX loss. After adjusting for effects of age, surgery and pre-operative ADL statuses, only MGMT promoter methylation was found to be significantly associated with longer overall survival time in grade III (*p* = 0.024) and IV patients (*p* = 0.006).

**Conclusions:**

The incidences of MGMT promoter methylation and IDH1 mutation were found to be comparable to globally reported rates, but those of 1p19q co-deletion and ATRX loss seemed to be lower in our cohort. MGMT promoter methylation was associated with increased overall survival in our cohort and might serve as favorable prognostic factor.

## Background

Gliomas are the most prevalent primary brain malignancy, accounting for more than 80% of primary brain tumors [1]. They are a highly heterogeneous group of tumors arising from glial cells in the central nervous system [2]. In their most aggressive form that is glioblastoma, prognosis is dismal with the median survival being less than two years despite maximal surgical resection and adjuvant chemoradiotherapy [3–5]. One of the contributors to such poor outcomes is the molecular heterogeneity of gliomas [6], which makes treatment challenging. Consequently, there has been a move towards molecular profiling of these tumors, in the hope of providing personalized precision treatment in order to improve the overall survival and quality of life of patients afflicted with this devastating disease.

There have been significant advances made in the classification of brain tumors over the last decade, with the introduction of the molecular-based 2016 World Health Organization (WHO) Classification of Tumors of the Central Nervous System [7]. The use of molecular genotypes and phenotypes in the 2016 classification accorded a degree of objectivity not previously present in the 2007 classification [8], which was primarily based on microscopic characteristics of tumor cells relative to their cells of origin and levels of differentiation. Molecular signatures of gliomas have shown that histologically distinct tumor subtypes might share common precursor cells, while histologically indistinguishable gliomas could be separated into biologically and molecularly distinct classes [9]. These molecular markers might also serve as prognostic and predictive markers, indicators of disease aggressiveness and treatment response, and potential therapeutic targets [10, 11]. Examples include hypermethylation of O^6^-methylguanine-DNA-methyltransferase (MGMT), 1p19q co-deletion, isocitrate dehydrogenase (IDH) gene mutations 1 and 2, and inactivating mutations of alpha-thalassemia/mental retardation syndrome X-linked (ATRX).

Knowledge of the incidences of significant diagnostic and prognostic biomarkers in high-grade gliomas, as well as their impact on survival in various populations would allow for further research into the pathogenicity of such gliomas and lead to more targeted therapeutics. This study aimed to report the incidences of four molecular biomarkers in high-grade gliomas in an ethnically diverse Southeast Asian population, which have thus far not been reported. The impact of these biomarkers on overall survival in high-grade gliomas would also be investigated.

## Methods

### Study design

We conducted a retrospective review of patients who underwent biopsy or surgical resection of cerebral tumors from January 2013 to March 2017 at National Neuroscience Institute. The inclusion criteria were patients aged 21 years and above at time of surgery: a histological diagnosis of grade III or grade IV glioma, and at least one biomarker included in the histological report. All patients who underwent surgical treatment had maximal safe surgical resection followed by a standard post-surgical treatment routine consisting of combined chemoradiation (Stupp protocol) whenever feasible. The post-treatment plan was determined by a multidisciplinary team to ensure consistent treatment. Patients without any molecular biomarker testing were excluded. A total of 400 patients underwent surgery from January 2013 to March 2017. A total of 181 patients were selected, of whom 56 had grade III gliomas and 125 had grade IV gliomas. All patients in the study were followed up till January 2019 or till death, whenever earlier. Their records from hardcopy case notes and electronic databases were reviewed. This retrospective study was approved by the SingHealth Centralized Institutional Review Board.

### Data collection

Patient variables recorded include age at time of surgery, sex, ethnicity, Karnofsky Performance Status Scale (KPS) score, ability to perform activities of daily living (ADLs), tumor location and biomarkers status. Patients were divided into five ethnic groups: Chinese, Indian, Malay, Caucasian and others, which included Polynesians and Africans. The dates of death for patients who died during the study period were obtained from the Registry of Births and Deaths in the Immigration and Checkpoints Authority (Singapore) via the National Records of Diseases. Overall survival was defined as the time from first diagnosis via histologic confirmation until death or last follow-up.

The presence of biomarkers was defined as: methylation of the MGMT promoter, presence of co-deletion of chromosomes 1p and 19q, mutation of IDH1 gene and loss of ATRX staining. The testing of biomarkers was done using a combination of techniques, namely methylation-specific polymerase chain reaction and capillary electrophoresis for MGMT methylation, immunohistochemistry for IDH1 and ATRX staining, and fluorescence in-situ hybridization (FISH) for 1p19q co-deletion. Details on these techniques and examples of such stains have been well-described in several papers [12, 13]. Biomarkers detection was performed on histological specimens obtained at the time of surgery prior to treatment, except for cases of recurrent gliomas, for which these patients had prior adjuvant chemoradiation therapy.

### Statistical analysis

Descriptive data were expressed as means ± standard deviations, or medians (interquartile range (IQR)). Two-sided Chi-square with continuity correction and two-sided Mann-Whitney U tests were used to compare categorical and continuous variables respectively. A *p* value of < 0.05 was considered statistically significant. The median follow-up time was estimated using the reverse Kaplan-Meier method [14], where being alive was treated as the event of interest and death was censored. Lengths of survival were represented as medians (95% confidence intervals (CI)). Survival curves were plotted using the Kaplan-Meier method and compared using the log-rank test. Kaplan-Meier survival analysis was not performed for 1p19q co-deletion in grade IV gliomas as only one patient tested positive. Survival curves for ATRX loss in both groups were not evaluated as none tested positive in the grade III group while only two tested positive in the grade IV group. Univariate Cox regression analysis was performed to explore the predictive roles of the biomarkers for survival time. Multivariable Cox regression analysis was used to assess the predictive role of various biomarkers after adjusting for other potential predictors with *p* < 0.05 from univariable analysis. Statistical analysis was performed using SPSS (version 22.0).

## Results

### Patient demographics

The demographic and clinical data of all patients are summarized in Table 1. There were no significant differences between the grade III and grade IV glioma groups except for age. Patients in the grade III glioma group (median age 50 years, IQR 38–64) were significantly younger than grade IV glioma patients (59 years, IQR 44–67) (*p* = 0.017). Out of 181 cases, 19% of patients had recurrent gliomas (12 grade III, 22 grade IV). Surgical tumor resection was performed for 85% (45 (80%) grade III, 109 (87%) grade IV) with the remaining 15% undergoing biopsy alone. The overall median follow-up duration was 68.7 (95% CI: 62.1–75.3) months.

**Table 1:** Baseline characteristics of patient cohort

Variables	Overall	Grade III	Grade IV	*p*
n (%)	181	56 (31)	125 (69)	–
Male, n (%)	111 (61)	31 (55)	80 (64)	0.348
Ethnicity, n (%)				0.554
Chinese	126 (70)	41 (73)	85 (68)	–
Malay	26 (14)	9 (16)	17 (13)	–
Indian	20 (11)	4 (7)	16 (13)	–
Caucasian	7 (4)	2 (4)	5 (4)	–
Others	2 (1)	0	2 (2)	–
Median (*IQR*) age	57 (43–66)	50 (38–64)	59 (44–67)	**0.017**
Median (*IQR*) preop KPS	80 (70–80)	80 (70–80)	80 (70–80)	0.730
Preop ADL-independent, n (%)	159 (88)	49 (88)	110 (88)	0.924
Tumor Location, n (%)				0.499
Left hemisphere	85 (47)	26 (46)	59 (47)	–
Right hemisphere	92 (51)	28 (50)	64 (51)	–
Cerebellar	3 (2)	2 (4)	1 (1)	–
Spinal	1 (1)	0	1 (1)	–
Recurrent case, n (%)	34 (19)	12 (21)	22 (18)	0.686
Surgery type, n (%)				0.333
Biopsy	27 (15)	11 (20)	16 (13)	–
Resection	154 (85)	45 (80)	109 (87)	–

*IQR* interquartile range; *KPS* Karnofsky performance scale; *ADL* Activities of daily living. For all variables (with the exception of age and KPS), *P*-values were calculated from two-sided Chi-square statistics with Yates correction to compare the presence or absence of the specific biomarker in grade III versus grade IV gliomas. *P*-values were calculated from two-sided Mann-Whitney U test to compare the median age and KPS scores between grade III versus grade IV gliomas

Figures in boldface represent *p* values of less than 0.05

### Incidence of biomarkers within the population

In our study cohort, 94 patients were tested for MGMT promoter methylation, 92 for 1p19q co-deletion, 102 for IDH1 mutation and 32 for ATRX loss (see Table 2). Higher percentages of biomarkers were observed in grade III compared to grade IV patients for MGMT promoter methylation (55% versus 30%), 1p19q co-deletion (41% versus 2%), and IDH1 mutation (35% versus 15%) with the exception of ATRX loss (0% versus 8%).

**Table 2:** Incidence of biomarkers

Biomarker	Overall	Grade III	Grade IV	*p*
n	181	56	125	–
MGMT (n tested)	94	31	63	**0.037**
Methylated, n (%)	36 (38)	17 (55)	19 (30)	–
Non-methylated, n (%)	58 (62)	14 (45)	44 (70)	–
1p19q co-deletion (n tested)	92	39	53	**< 0.01**
Present, n (%)	17 (18)	16 (41)	1 (2)	–
Absent, n (%)	75 (82)	23 (59)	52 (98)	–
IDH1 mutation (n tested)	102	23	79	**0.074**
Present, n (%)	20 (20)	8 (35)	12 (15)	–
Absent, n (%)	82 (80)	15 (65)	67 (85)	–
ATRX (n tested)	32	7	25	0.440
ATRX loss, n (%)	2 (6)	0	2 (8)	–
ATRX intact, n (%)	30 (94)	7 (100)	23 (92)	–

*MGMT* O^6^-methylguanine-DNA-transferase; *IDH1* isocitrate dehydrogenase 1; *ATRX* alpha-thalassemia/mental retardation syndrome X-linked. *P*-values were calculated from two-sided Chi-square statistics with Yates correction to compare the presence or absence of the specific biomarker in grade III versus grade IV gliomas

Figures in boldface represent *p* values of less than 0.05

### Incidence of biomarkers within different ethnic groups

The biomarkers incidences across various ethnic groups were examined (see Online Resources 1 and 2). The incidences of all four biomarkers among the Chinese were similar to those of the overall population for both grade III and grade IV gliomas except for a lower incidence of 1p19q co-deletion among Chinese grade III glioma patients (31% vs 41% overall). In this group, 7 of the 16 grade III glioma patients who tested positive for 1p19q co-deletion were non-Chinese: 4 Malays (67%), 1 Indian (50%) and 2 Caucasians (100%). In the grade IV glioma group, the only patient who tested positive for the 1p19q co-deletion was a Caucasian.

Subgroup analysis by ethnicity in the grade III glioma group showed that Malays had the highest incidence of positive results for two biomarkers: 60% for MGMT promoter methylation and 67% for 1p19q co-deletion. For the grade IV glioma group, Indians were found to have the highest incidence of positive results for two of the biomarkers: MGMT promoter methylation (50%) and IDH1 mutation (30%). The two patients who tested positive for ATRX loss in the grade IV glioma group were both Chinese.

### The impact of biomarkers on survival outcomes

**Fig. 1:**
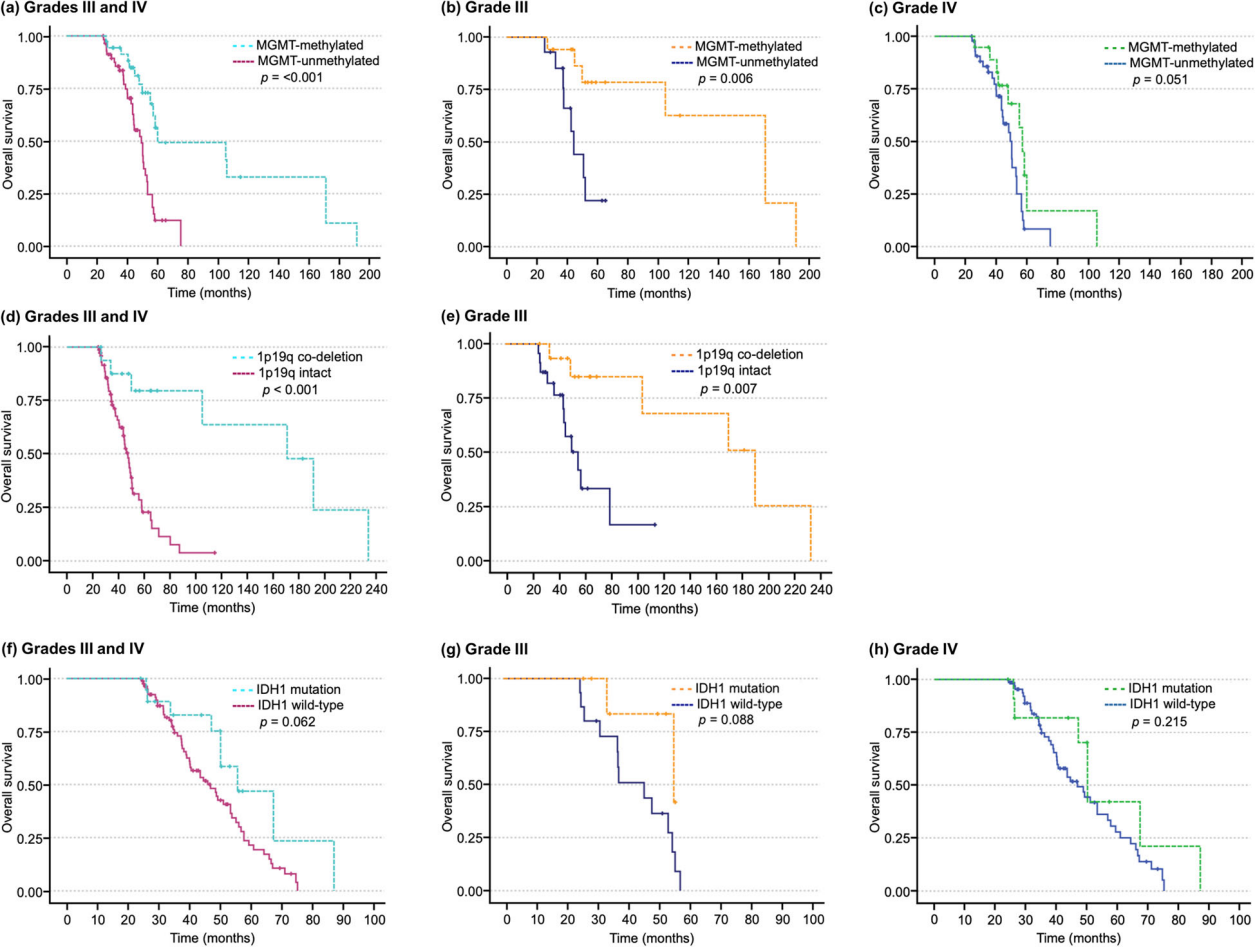
Kaplan-Meier survival curves for various biomarkers

Survival curves for the various glioma biomarkers are shown in Fig. 1. The median overall survival for grade III glioma patients with MGMT promoter-methylation was 171.0 (95% CI: 115.4–226.6) months compared to just 44.3 (95% CI: 39.3–49.3) months in those without the mutation (*p* = 0.006). Grade III glioma patients with 1p19q co-deletion had a median overall survival of 191.4 (95% CI: 109.6–273.2) months compared to 55.7 (95% CI: 40.0–71.4) months for those who tested negative (*p* = 0.007). There was no significant difference in median survival for IDH1 mutation in grade III gliomas although there was a trend towards improved overall survival in those who harbored the mutation (*p* = 0.088). Univariable cox regression identified the presence of MGMT promoter methylation (*p* = 0.013), 1p19q co-deletion (*p* = 0.014), younger age and surgical excision as potential predictors of longer survival in grade III gliomas. IDH1 mutation (*p* = 0.11) did not significantly predict survival time. MGMT (*p* = 0.024), but not 1p19q (*p* = 0.094) or IDH1 (*p* = 0.77), remained a significant predictor of survival time after adjusting for age at diagnosis and surgical treatment.

The median overall survival for grade IV glioma patients with MGMT promoter-methylation was 57.1 (95% CI: 52.8–45.6) months compared to 50.2 (95% CI: 44.1–56.2) months in those without the mutation (*p* = 0.051). Grade IV glioma patients with IDH1 mutation had a median overall survival of 46.9 (95% CI: 38.2–55.5) months compared to 50.2 (95% CI: 46.5–53.9) months for those who tested negative (*p* = 0.22). Univariable Cox regression identified younger age (*p* = 0.004), preoperative ADL (*p* = 0.018) and surgical excision (*p* = 0.019) as potential predictors of longer survival in grade IV gliomas. The presence of MGMT promoter methylation (*p* = 0.006), but not IDH1 mutation (*p* = 0.81), was a significant predictor of survival time after adjusting for age at diagnosis, preoperative ADLs and surgical treatment.

## Discussion

This study investigated the incidences of four molecular biomarkers, namely MGMT promoter methylation, 1p19q co-deletion, IDH1 mutation and ATRX loss, in a Southeast Asian population. Our study cohort reflected the unique heterogeneity of an ethnically diverse population, made up predominantly of ethnic Chinese (74.3% of the population), Malays (13.4%) and Indians (9.0%) [15]. To the best of our knowledge, the incidences of high-grade gliomas and associated biomarkers in such a population have not been reported.

The MGMT promoter methylation is a loss-of-function mutation that plays an important early role in tumorigenesis [16]. In our study, the incidence of MGMT promoter methylation in high-grade gliomas was 55% in grade III gliomas and 30% in grade IV gliomas. Three studies done in China in 2011 [17], 2015 [18] and 2016 [19] placed the incidence of these mutations in glioblastomas at 33, 40 and 30% respectively, similar to the incidence of 32% in the ethnic Chinese in our study. In a Japanese study [20], the incidence of MGMT promoter methylation was 36% in grade III gliomas and 46% in glioblastomas. India reported one of the highest incidences of these mutations - 81% for grade III gliomas [21] and 63% for grade IV glioblastomas [22]. As for Caucasian-based studies, the reported incidence ranged from 35 to 45% in high-grade gliomas [23]. A Dutch study in 2013 [24] found the incidence of such mutations to be 45% in anaplastic gliomas and 27% in glioblastomas. Studies from Australia [25], France [26] and Italy [27] placed the incidence of these mutations in grade IV gliomas to range from 48 to 57%. While the MGMT promoter methylation incidence in our cohort lay within the range quoted by global studies (Tables 3 & 4), further studies are required to investigate intra-ethnic variations within the population.

**Table 3:** Incidence of biomarkers in grade III gliomas in other studies

Study	Year	Country	Number of patients tested	Incidence of Positive Biomarkers (%)			
				MGMT promoter methylation	1p19q co-deletion	IDH1 mutation	ATRX loss
Lassman et al. [28]	2011	USA	631		48		
Boots-Sprenger et al. [24]	2013	Netherlands	51 (MGMT)	45			
			53 (1p19q)		51		
			115 (IDH1)			75	
Wiestler et al. [29]	2013	Germany	133				33
Ogura et al. [20]	2015	Japan	101	36		35	
Cai et al. [30]	2016	China	104			54	40
Ebrahimi et al. [31]	2016	Germany	245 (IDH1/2)			60^a^	
			239 (ATRX)				34
Polivka et al. [32]	2016	Czech Republic	23		52		
Rajmohan et al. [21]	2016	India	91	81	53	84	30
Kramář et al. [33]	2016	Czech Republic	17			71^a^	

^a^Includes IDH2 mutations. *MGMT* O^6^-methylguanine-DNA-transferase; *IDH1* isocitrate dehydrogenase 1; *ATRX* alpha-thalassemia/mental retardation syndrome X-linked

**Table 4:** Incidence of biomarkers in grade IV gliomas in other studies

Study	Year	Country	Number of patients tested	Incidence of Positive Biomarkers (%)			
				MGMT promoter methylation	1p19q co-deletion	IDH1 mutation	ATRX loss
Tang et al. [17]	2011	China	79	33			
Lechapt-Zalman et al. [26]	2012	France	110	57			
Nehru et al. [22]	2012	India	27	63			
Boots-Sprenger et al. [24]	2013	Netherlands	321 (MGMT)	27			
			325 (1p19q)		3		
			223 (IDH1)			16	
McDonald et al. [25]	2015	Australia	33	48			
Ogura et al. [20]	2015	Japan	165	46		4	
Yang et al. [18]	2015	China	238 (MGMT)	40			
			260 (IDH1/2)			21^a^	
Cai et al. [30]	2016	China	114			15	12
Chaurasia et al. [34]	2016	Korea	163			10	15
Ebrahimi et al. [31]	2016	Germany	243 (IDH1/2)			7^a^	
			242 (ATRX)				11
Kramář et al. [33]	2016	Czechoslovakia	58			17^a^	
Li et al. [19]	2016	China	145	30		17	
Tini et al. [27]	2016	Italy	169	50			

^a^Includes IDH2 mutations. *MGMT* O^6^-methylguanine-DNA-transferase; *IDH1* isocitrate dehydrogenase 1; *ATRX* alpha-thalassemia/mental retardation syndrome X-linked

The 1p19q co-deletion was initially described in 1994 [35] and has been recognized as a hallmark molecular signature of oligodendrogliomas. In our cohort, 41% of grade III gliomas harbored the 1p19q co-deletion, comparatively lower than those quoted by global studies, most of which were done in predominantly Caucasian populations. A Czech study [32] reported a 52% incidence of 1p19q co-deletions in anaplastic oligodendrogliomas, while three other studies from America [28], Netherlands [24] and India [21] also reported this mutation to be present in 48, 51 and 53% of grade III gliomas respectively. 2% of our grade IV glioma patients tested positive for 1p19q co-deletion, which was close to the reported rate of 3% in the Dutch study [24].

Like MGMT promoter methylation, IDH1 mutations are considered an early event in gliomagenesis [36]. They are commonly associated with lower-grade gliomas (WHO grades II and III) and secondary glioblastomas, occurring in more than 80% of such tumors [37]. In our cohort, 35% of grade III glioma patients had IDH1 mutation and the global incidence varies according to region (Table 3). Boots-Sprenger et al. [24] cited 75% of anaplastic gliomas with IDH1 mutation in the Netherlands while Ogura et al. [20] and Rajmohan’s group [21] cited contrasting incidences of 35% in Japan and 84% in India respectively. On the other hand, the incidence of IDH mutations in primary glioblastomas is much lower (Table 4). Parsons and colleagues were one of the first to discover recurrent IDH mutations in 12% of primary glioblastomas [38]. Since then, multiple studies from various different regions including Korea [34], China [18, 19, 30], Czech Republic [33] and Netherlands [24] have quoted the incidence of IDH mutations in primary glioblastomas to range between 10 and 20%. The incidence of IDH1 mutations in grade IV gliomas in our cohort was 15%.

Compared to the previous three biomarkers, the ATRX gene is relatively new to the glioma scene. It was initially discovered in patients with the alpha-thalassemia X-linked mental retardation syndrome (ATRX syndrome) [39] and plays a crucial role in maintaining genomic stability [40–42]. The incidence of ATRX loss in high-grade gliomas varies across regions (Tables 3 & 4). In grade III gliomas, its incidence was quoted as 30% in an Indian population [21], 33% in a German study [29] and 40% in a Chinese population [30]. In glioblastomas, the incidence of ATRX loss ranged between 10 and 15%, specifically 11% in Germany [31], 12% in China [30] and 15% in Korea [34]. Compared to these studies, our incidence of ATRX loss was lower at 8% in grade IV gliomas, while none of the 7 grade III gliomas tested in our cohort returned positive for this mutation.

This study showed that with the exception of IDH1 mutations, our incidences of 1p19q co-deletion and ATRX loss appeared to be lower than globally reported rates while our incidence of MGMT promoter methylation was towards the lower end of the range quoted by global studies (Tables 3 & 4). This suggests that our patients with high-grade gliomas may have distinct genetic and molecular signatures as compared to those from other countries. Studies [43–45] have shown that ethnic differences could contribute to inherited susceptibility to primary malignant gliomas, pointing to distinct and separate genetic pathways of tumorigenesis involving p53 and PTEN (phosphatase and tensin homologue deleted from chromosome 10) genes in different racial groups, though there have been no reported studies on our four biomarkers. Environmental risk factors could also possibly contribute to the differing incidences of the various biomarkers across geographical regions, although there have been few conclusive studies [46]. More in-depth research into the roles of genetic and environmental risk factors in the development of malignant gliomas would allow us to tailor treatment and prognostication models in different populations.

We also explored the impact of these biomarkers on overall survival in our population and found that longer overall survival was associated with the presence of MGMT promoter methylation (in grade III and IV) and 1p19q co-deletion (in grade III glioma only). These findings appeared to be in line with the NOA-4 study [47] and the study by van den Bent et al. [48], both of which showed that MGMT promoter methylation appeared to be more of an independent prognostic factor rather than a predictive factor for treatment response in grade III anaplastic oligodendrogliomas. Bell et al. [49] also echoed similar findings in a more recent study involving anaplastic astrocytomas treated with radiation plus nitrosourea or radiation plus temozolamide. These suggest that the favorable outcomes observed in MGMT-methylated grade III gliomas seem to be irrespective of treatment regimes [9]. In contrast, the clinical prognostic value of MGMT promoter methylation in grade IV gliomas remained unclear, although its significance as a predictor of treatment outcome to combined chemoirradiation with temozolamide in glioblastomas has been demonstrated in some studies [23]. A recent meta-analysis [50] found that prolonged overall survival in glioblastoma patients was accompanied by MGMT promoter methylation in European and American populations but this was not the case in the Asian group. Further studies are required to elucidate the varying clinical implications of MGMT promoter methylation on treatment and survival in different ethnic and geographical populations. For 1p19q co-deletion, while its exact biologic effect in gliomas is not clear, its presence has been associated with increased chemosensitivity and hence, a more favorable prognosis [51, 52]. This association was evident in our grade III glioma patients harbouring this mutation. Polivka et al. [27], likewise, observed that 1p19q co-deletion served as a strong prognostic and predictive biomarker for patients with anaplastic oligodendrogliomas.

Patients with IDH-mutant gliomas have been shown to have better prognosis than those with wild-type IDH regardless of glioma grade or histology [53, 54]. Our results did not show any significant survival benefit in IDH-mutant gliomas for both grade III and grade IV tumours. This may be due to the fact that IDH mutations are more commonly found in lower-grade gliomas and secondary glioblastomas, both of which were not included in our study population.

As the incidence of ATRX loss in our population is low, we are unable to draw any conclusions about the prognostic significance of this biomarker. However, it has been shown that ATRX loss is often associated with IDH mutations, but rarely with 1p19q co-deletions [41]. ATRX loss may also help define a subset of IDH-mutant gliomas with a significantly longer median time to treatment failure [29, 55]. In fact, a study by Mukherjee et al. suggested that mutant IDH may work synergistically with ATRX loss to drive alternative lengthening of telomere phenotype in gliomas, hence conferring a survival advantage in this subset of glioma patients [56].

This study is unique because of its ethnically diverse and heterogeneous population. Moreover, only 7% (12/181) of our study population was lost in the follow-up process. However, it has a few limitations. The decision for genetic profiling and choice of biomarkers to be tested were made at the clinicians’ discretion, hence not all patients were tested for all the biomarkers. Biomarkers detection techniques also varied among different centers. In addition to the small sample size, the low event rate (presence of biomarkers) and selection bias might have affected the power of the study. Results should therefore be interpreted with caution. We acknowledge that the study may not be sufficiently powered to detect potential associations of biomarkers and survivals with Cox regression. Future larger studies are required to validate the findings from this study. Another limitation is the lack of detailed clinical records regarding the chemotherapy and radiotherapy regimes that the patients underwent. This information would allow us to analyze the survival outcomes in relation to various treatment regimes.

## Conclusion

With the introduction of the 2016 revised WHO Classification of Tumors of the Central Nervous System, molecular markers have becoming increasingly important in the diagnosis, treatment and prognostication of gliomas. In this study, our incidences of MGMT promoter methylation and IDH1 mutation were comparable to globally reported rates while those for 1p19q co-deletion and ATRX loss in our population were lower. There appears to be survival benefit for patients with MGMT promoter methylation in both grade III and IV patients, and 1p19q co-deletion in grade III glioma. MGMT appeared to carry greater prognostic value in our patients for grade III and IV glioma patients.

## Supplementary information

**Additional file 1.** Online Resource 1 Incidence of biomarkers across different ethnicities for Grade III gliomas.

**Additional file 2.** Online Resource 2 Incidence of biomarkers across different ethnicities for Grade IV gliomas.

## Data Availability

The datasets generated during and/or analysed during the current study are available from the corresponding author on reasonable request.

## Abbreviations

ADL: Activities of daily living
ATRX: Alpha-thalassemia/mental retardation syndrome X-linked
CI: Confidence interval
FISH: Fluorescence in-situ hybridization
IDH: Isocitrate dehydrogenase
IQR: Interquartile range
MGMT: O^6^-methylguanine-DNA-methyltransferase
WHO: World Health Organization
