# Supplementary material for: Incidence of biomarkers in high-grade gliomas and their impact on survival in a diverse SouthEast Asian cohort - a population-based study
Source: BMC Cancer. 2020 Jan 31;20:79. doi: 10.1186/s12885-020-6536-x (PMC6993394; doi:10.1186/s12885-020-6536-x)
Supplement: Supplementary file 1 — Additional file 1. Online Resource 1 Incidence of biomarkers across different ethnicities for Grade III gliomas. [file 12885_2020_6536_MOESM1_ESM.docx]

**Online Resource 1** Incidence of biomarkers across different ethnicities for Grade III gliomas

| Biomarker | Overall | Chinese | Malay | Indian | Caucasian | Others | *p* |  |
| --- | --- | --- | --- | --- | --- | --- | --- | --- |
| MGMT (n tested) | 31 | 22 | 5 | 2 | 2 | 0 | 0.248 |  |
| Methylated, n (%) | 17 (55) | 12 (55) | 3 (60) | 0 | 2 (100) | 0 | - |  |
| Non-methylated, n (%) | 14 (45) | 10 (45) | 2 (40) | 2 (100) | 0 | 0 | - |  |
| 1p19q co-deletion (n tested) | 39 | 29 | 6 | 2 | 2 | 0 | 0.123 |  |
| Present, n (%) | 16 (41) | 9 (31) | 4 (67) | 1 (50) | 2 (100) | 0 | - |  |
| Absent, n (%) | 23 (59) | 20 (69) | 2 (33) | 1 (50) | 0 | 0 | - |  |
| IDH1 mutation (n tested) | 23 | 18 | 1 | 3 | 1 | 0 | 0.147 |  |
| Present, n (%) | 8 (35) | 6 (33) | 1 (100) | 0 | 1 (100) | 0 | - |  |
| Absent, n (%) | 15 (65) | 12 (67) | 0 | 3 (100) | 0 | 0 | - |  |
| ATRX (n tested) | 7 | 6 | 1 | 0 | 0 | 0 | N/A |  |
| ATRX loss, n (%) | 0 | 0 | 0 | 0 | 0 | 0 | - |  |
| ATRX intact, n (%) | 7 (100) | 6 (100) | 1 (100) | 0 | 0 | 0 | - |  |
| MGMT = O^6^-methylguanine-DNA-transferase; IDH1 = isocitrate dehydrogenase 1; ATRX = alpha-thalassemia/mental retardation syndrome X-linked | | | | | | | | |
