# Supplementary material for: Incidence of biomarkers in high-grade gliomas and their impact on survival in a diverse SouthEast Asian cohort - a population-based study
Source: BMC Cancer. 2020 Jan 31;20:79. doi: 10.1186/s12885-020-6536-x (PMC6993394; doi:10.1186/s12885-020-6536-x)
Supplement: Supplementary file 2 — Additional file 2. Online Resource 2 Incidence of biomarkers across different ethnicities for Grade IV gliomas. [file 12885_2020_6536_MOESM2_ESM.docx]

**Online Resource 2** Incidence of biomarkers across different ethnicities for Grade IV gliomas

| Biomarker | Overall | Chinese | Malay | Indian | Caucasian | Others | *p* |  |
| --- | --- | --- | --- | --- | --- | --- | --- | --- |
| MGMT (n tested) | 63 | 41 | 11 | 8 | 2 | 1 | 0.465 |  |
| Methylated, n (%) | 19 (30) | 13 (32) | 2 (18) | 4 (50) | 0 | 0 | - |  |
| Non-methylated, n (%) | 44 (70) | 28 (68) | 9 (82) | 4 (50) | 2 (100) | 1 (100) | - |  |
| 1p19q co-deletion (n tested) | 53 | 35 | 8 | 6 | 2 | 2 | **<0.001** |  |
| Present, n (%) | 1 (2) | 0 | 0 | 0 | 1 (50) | 0 | - |  |
| Absent, n (%) | 52 (98) | 35 (100) | 8 (100) | 6 (100) | 1 (50) | 2 (100) | - |  |
| IDH1 mutation (n tested) | 79 | 55 | 8 | 10 | 4 | 2 | 0.432 |  |
| Present, n (%) | 12 (15) | 8 (14) | 0 | 3 (30) | 1 (25) | 0 | - |  |
| Absent, n (%) | 67 (85) | 47 (86) | 8 (100) | 7 (70) | 3 (75) | 2 (100) | - |  |
| ATRX (n tested) | 25 | 17 | 3 | 3 | 1 | 1 | 0.906 |  |
| ATRX loss, n (%) | 2 (8) | 2 (12) | 0 | 0 | 0 | 0 | - |  |
| ATRX intact, n (%) | 23 (92) | 15 (88) | 3 (100) | 3 (100) | 1 (100) | 1 (100) | - |  |
| MGMT = O^6^-methylguanine-DNA-transferase; IDH1 = isocitrate dehydrogenase 1; ATRX = alpha-thalassemia/mental retardation syndrome X-linked | | | | | | | | |
